# Prevalence of type-1 interferon autoantibodies in adults with non-COVID-19 acute respiratory failure

**DOI:** 10.1186/s12931-022-02283-4

**Published:** 2022-12-16

**Authors:** Rajani Ghale, Natasha Spottiswoode, Mark S. Anderson, Anthea Mitchell, Grace Wang, Carolyn S. Calfee, Joseph L. DeRisi, Charles R. Langelier

**Affiliations:** 1 Division of Pulmonary, Critical Care, Allergy, and Sleep Medicine, University of California, San Francisco, CA USA; 2 Division of Infectious Diseases, University of California, San Francisco, CA USA; 3 Department of Medicine, University of California, San Francisco, CA USA; 4 Diabetes Center, University of California, San Francisco, CA USA; 5 Department of Biochemistry and Biophysics, University of California, San Francisco, CA USA; 6 Chan Zuckerberg Biohub, San Francisco, CA USA

**Keywords:** COVID-19, Auto-antibodies, Type I interferon, Anti-interferon antibodies, Acute respiratory failure

## Abstract

Auto-antibodies (Abs) to type I interferons (IFNs) are found in up to 25% of patients with severe COVID-19, and are implicated in disease pathogenesis. It has remained unknown, however, whether type I IFN auto-Abs are unique to COVID-19, or are also found in other types of severe respiratory illnesses. To address this, we studied a prospective cohort of 284 adults with acute respiratory failure due to causes other than COVID-19. We measured type I IFN auto-Abs by radio ligand binding assay and screened for respiratory viruses using clinical PCR and metagenomic sequencing. Three patients (1.1%) tested positive for type I IFN auto-Abs, and each had a different underlying clinical presentation. Of the 35 patients found to have viral infections, only one patient tested positive for type I IFN auto-Abs. Together, our data suggest that type I IFN auto-Abs are uncommon in critically ill patients with acute respiratory failure due to causes other than COVID-19.

**Supplementary Information:**

The online version contains supplementary material available at 10.1186/s12931-022-02283-4.

**Correspondence**

Type I interferon (IFN) signaling is an essential component of antiviral innate immunity, and may be impaired in patients with severe COVID-19 [1–4]. Auto-antibodies (Abs) to type I interferons (IFNs) are found in up to 25% of patients with severe COVID-19, but rarely in mild COVID-19 or in healthy controls [1–4], suggesting a decided role in disease pathogenesis. This is further supported by studies demonstrating that patients with autoimmune polyendocrine syndrome type I (APS-1), which leads to type I IFN auto-Ab production in childhood, are predisposed to severe COVID-19 [5].

While several compelling studies have now established a link between type I IFN auto-Abs and COVID-19 severity, it has remained unknown whether their association with severe disease is unique to COVID-19 or common to other forms of severe respiratory illness. To address this question, we studied a prospective cohort of adults without COVID-19 admitted to the intensive care unit (ICU) for acute respiratory failure requiring mechanical ventilation, and measured type I IFN auto-Abs. Clinical and demographic features of the cohort are described in (Table 1). Causes of acute respiratory failure included viral or bacterial pneumonia, non-pulmonary sepsis, stroke or other acute neurologic process, cardiogenic edema, surgical complication, cardiac arrest, and others (Additional file 1: Table S1). Subjects were enrolled between 7/2013 and 3/2020 under University of California San Francisco (UCSF) Institutional Review Board protocol #17-24056.

**Table 1:** Clinical and demographic features of cohort

Age (median, Q1–Q3)	61 (49–70)
Gender (median, %)	
Male	179, 63%
Female	105, 37%
Race (n, %)	
Caucasian	147, 52%
Asian	53, 19%
African American	17, 6%
Pacific Islander	7, 2%
Native American	2, 1%
Other	55, 19%
Unknown	3, 1%
Ethnicity (n, %)	
Non-LatinX	231, 81%
LatinX	53, 19%
Temp Max (median, Q1–Q3)	37.7 (37.2–38.2)
WBC Max (median, Q1–Q3)	12.6 (8.2–17.3)
Pneumonia (n, %)	107, 38%
Viral pneumonia (n, %)	35, 12%
28-day mortality (n, %)	71, 25%
Intubated (n, %)	284, 100%
Vasopressors (n, %)	178, 63%
*Immunocompromised (n, %)	48, 17%

*Solid organ transplant, hematopoietic stem cell transplant, HIV infection, leukemia, lymphoma

Auto-Abs to IFN-a2 were measured from plasma collected within 72 h of intubation using a radioligand binding assay (RLBA), according to previously developed methods [2]. A positive signal was defined as greater than 2 standard deviations above the mean of pre–COVID-19 blood bank healthy controls (n = 18). Three patients with APS-1 were also included as positive controls. Subjects with pneumonia were identified using a previously described adjudication protocol [6], and screened for viral pathogens by clinical respiratory viral PCR testing, SARS-CoV-2 PCR for those enrolled after 01/2020, and metagenomic next generation RNA sequencing, following established protocols [6].

Of the 284 subjects with acute respiratory failure, only three (1.1%) tested positive for type I IFN auto-Abs by RLBA (Fig. 1). These included a man with fatal rhinovirus pneumonia, a woman with post-operative complications of aneurysm repair and suspected thymoma, and a man with post-operative hemorrhage (Fig. 1). While each had different underlying clinical presentations, all were over the age of 67, which is associated with increased prevalence of type I IFN auto-Abs [3], and one had a probable thymoma, which is also associated with these auto-Abs [7]. With respect to non-COVID-19 viral pneumonia, one of 14 patients with rhinovirus infection had type I IFN auto-Abs (~ 7%); notably, this patient was also a bone marrow transplant recipient and had been diagnosed with graft-vs-host disease. For other viruses, including influenza (n = 11), parainfluenza (n = 6), metapneumovirus (n = 2), and seasonal coronavirus (n = 2), no positives were detected.

**Fig. 1:**
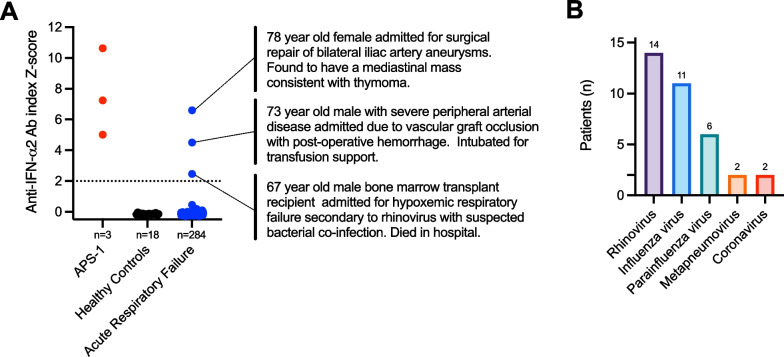
**A** Type I IFN auto-Ab detection by radioligand binding assay (RLBA) in adults with acute respiratory failure due to causes other than COVID-19 (blue), healthy control patients (black), or positive control patients with autoimmune polyglandular syndrome-1 (APS-1, red). Clinical features of the three patients with anti-type I IFN antibodies are described right of the panel. **B** Viruses detected in patients with pneumonia based a combination of multiplex respiratory viral PCR and metagenomic next generation RNA sequencing

Interferon signaling is a hallmark feature of viral respiratory infections including those due to influenza virus, SARS-CoV-2, and other pathogenic species [8, 9]. In contrast, our data demonstrate that detection of type I IFN auto-Abs is uncommon in critically ill patients with acute respiratory failure due to non-COVID-19 causes. This suggests that their association with pneumonia severity may have specificity for COVID-19, in line with the observation that auto-Abs to type I IFN are amongst the greatest risk factors for COVID-19 severity [3]. Given the relatively small numbers of distinct respiratory viral species represented in the cohort, we cannot exclude the possibility that type I IFN auto-Abs are associated with other severe viral pneumonias, although our results suggest an overall prevalence much lower than the 11–25% observed for patients with critical COVID-19 [1–4].

Strengths of our study include a large cohort size, detailed clinical phenotyping, and a prospective cohort of patients with acute respiratory failure from diverse causes. While our use of both clinical PCR testing and metagenomic sequencing for respiratory virus detection is also a strength, the relatively small number of confirmed respiratory viral infections is a limitation. Measurement of only a single type I IFN may also be a limitation, although prior work has demonstrated that IFN-a2 auto-Abs are most strongly associated with COVID-19 severity, and neutralize other type I IFNs, which somewhat alleviates this concern. We also recognize that factors such as timing and signaling magnitude have to be considered when evaluating the effects of interferon induction on target cells. Further studies are needed to assess the prevalence of auto-Abs against proteins other than type I IFNs in patients with critical illness from COVID-19 and other causes. Additional work is also needed to clarify the mechanisms underpinning the apparent specificity for type I IFN auto-antibodies in critical COVID-19 pneumonia.

## Supplementary Information

**Additional file 1: Table S1.** Source data for Fig. 1 including patient ID, RLBA Z-score versus pre–COVID-19 blood bank healthy controls, viruses detected, and etiology of respiratory failure for each patient.

## Data Availability

Source data are provided with this paper in Additional file 1: Table S1.
